# Cure of *mycobacterium avium* keratitis caused by trauma in elderly: case report

**DOI:** 10.3389/fcimb.2023.1268668

**Published:** 2023-09-21

**Authors:** Xiaoting Chen, Hongyan Wang, Kaizhen Wen, Shuilong Lin, Bingbing Li, Meiying Lin

**Affiliations:** ^1^ Medical Laboratory Center, Jinjiang Municipal Hospital, Jinjiang, Quanzhou, China; ^2^ Department of Ophthalmology, Jinjiang Municipal Hospital, Jinjiang, Quanzhou, China

**Keywords:** mycobacterium avium, keratitis, laboratory tests, non-tuberculous Mycobacteria (NTM), Mycobacterium morphology

## Abstract

We report a case of *Mycobacterium avium* keratitis, first detected in the laboratory, which is from an 81-year-old female patient with a 13-year history of recurrent keratitis after eye injuries. This patient underwent anterior chamber irrigation of the right eye, and autologous conjunctival flap covering plus medication, and the corneal ulcer healed. She recovered well after continuing with the medication for half a year. The patient was not immune-compromised. Complex eye diseases such as blurred vision and cataracts caused by advanced age, delayed symptoms caused by slow growth of *Mycobacterium avium* and low-grade inflammation, difficulty with laboratory culture, repeated medication use, and repeated illnesses were the main reasons for the prolonged failure to clarify the etiology of this case. Morphological examination provided important clues for the initial discovery of pathogenic bacteria. Remind to pay attention to the morphology of Mycobacterium under Gram staining and Rui’s Giemsa staining. Acid fast staining and Deoxyribonucleic Acid(DNA) microarray chip method can be used as indicators for rapid diagnosis of Mycobacterium species.

## Introduction

*Mycobacterium avium* complex (MAC) are the main members of non-tuberculous Mycobacteria (NTM), and they are the mycobacteria with the most new species and subspecies discovered. They are opportunistic pathogens that are ubiquitous in the environment. Infection rates have been rising in recent decades, and people with weakened immune systems are susceptible. Infection can affect a variety of tissues and organs, especially in the lungs, lymph nodes, skin, and soft tissue and in the form of disseminated disease and osteomyelitis. Eye infection is rarely reported. Ophthalmia is an inflammatory reaction that occurs inside and around the eyes which can be caused by both infectious and non-infectious factors. Infectious factors are associated with microbial infections, such as bacterial, viral, fungal and Rickettsia, chlamydia and so on. Non-infectious factors are usually caused by external stimuli such as smoke, dust, ultraviolet, trauma, acid-base burn. Part of ophthalmia are related to autoimmunity. The disseminated infections caused by MAC include ophthalmia in immunocompromised populations, such as those with acquired immunodeficiency syndrome, those positive for neutralizing antibodies for serum anti-interferon-γ (Ikeda et al., 2016), and those with interleukin (IL)-12 deficiency (Chong et al., 2015). In immunocompetent individuals, *Mycobacterium avium* intracellulare (MAI) keratitis is rare, and trauma and surgery are major risk factors (Knapp et al., 1987; Tyagi et al., 1999; Ko et al., 2017).

The clinical symptoms of mycobacterial keratitis are blurred vision with eye pain, photophobia, and redness (Knapp et al., 1987), (Ko et al., 2017), often mistaken for fungal, herpes simplex, or sterile inflammatory keratitis (Shah et al., 2016; Samuel et al., 1996; Ford et al., 1998). MAC are slow-growing mycobacteria. The slow process of infection and the delayed symptoms caused by low-grade inflammation reduce the clinical suspicion index. The bacteria require a long time and special conditions for culture, which may interfere with the diagnosis of the disease. The 81-year-old female patient in this article has a 13-year history of recurrent keratitis. The patient was diagnosed with *Mycobacterium avium* keratitis in June 2022 and underwent clinical treatment.

## Case introduction

**Figure 1:**
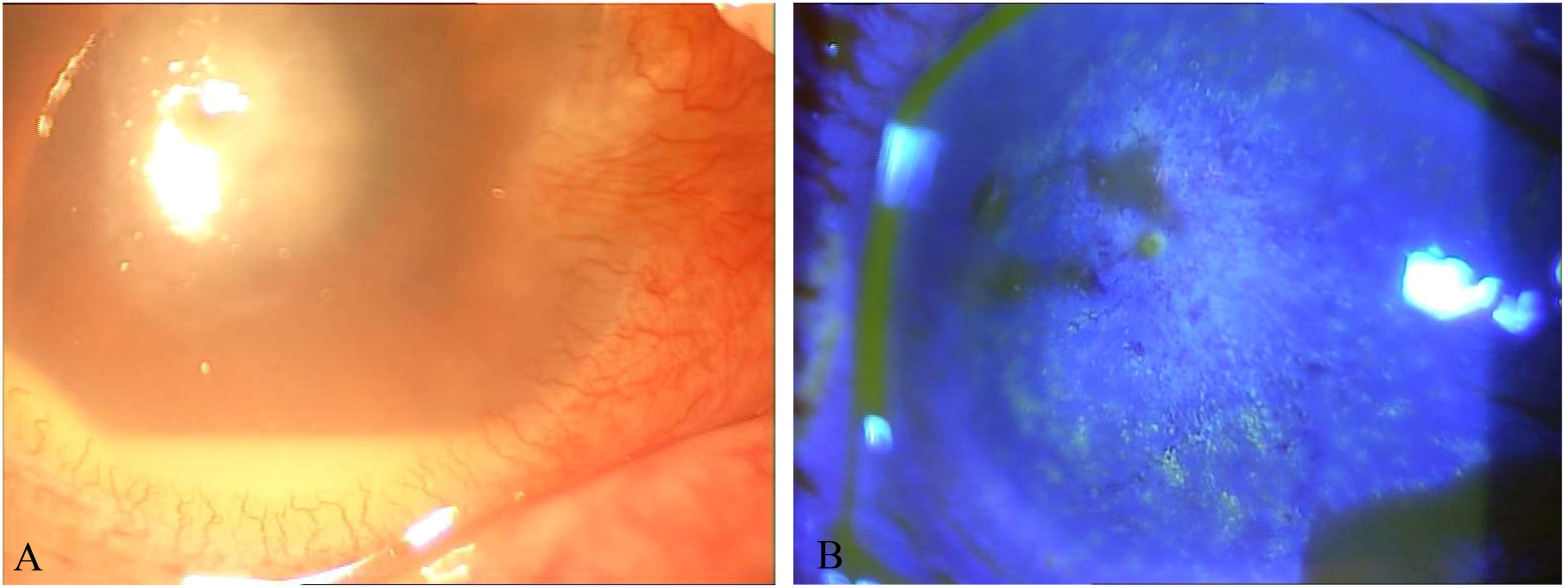
**(A)** Photo of the corneal ulcer in the right eye at initial diagnosis. **(B)** Photo of the corneal ulcer after fluorescein sodium staining at initial diagnosis.

The patient, female, 81 years old, came to Jinjiang Municipal Hospital with the main complaint of repeated redness and pain in the right eye caused by trauma, blurred vision for 13 years, two months ago, similar symptoms flared up again. In 2005, the patient’s right eye was accidentally injured by a farm implement (shovel), and she did not seek treatment. In May 2009, she first visited the doctor because of recurrent redness and pain in the right eye with blurred vision. The Best Corrected Visual Acuity(BCVA) vision was finger count for 50cm in 2009.The clinical diagnosis was keratitis in the right eye, complicated by cataract. Levofloxacin eye drops, Tobradex eye drops, Ganciclovir eye drops, and recombinant basic fibroblast growth factor eye drops alleviated the symptoms. In 2012, she went to the hospital again because the symptoms of red, pain, blurred vision in her right eye relapsed. At that time, the BCVA was 0.01. The clinical diagnosis were keratitis in the right eye, dry eye and cataract in both eyes. Cyclosporine eye drops, Tobradex eye drops, and Eyprotor eye gel eased her symptoms. After that, she was treated several times for recurring illnesses and came to our hospital with worsening illnesses 2 months ago. Eye examination showed light perception in positioning accuracy in the right eye and BCVA 0.12 in the left eye. Intraocular pressure: right 11.2 mmHg, left 13.5 mmHg (Non-contact tonometry method, NCT). The nasal conjunctiva of the left eye and its underlying connective tissue had abnormal hyperplasia, invading the corneal limbus for approximately 2 mm. Accord to Lens Opacities Classification System II(LOCS II), the lens wasC2N2P1. and the rest was normal. The conjunctiva of the right eye had mixed congestion, and the nasal conjunctiva and its underlying connective tissue had abnormal hyperplasia and invaded the corneal limbus by approximately 2 mm. The cornea was edematous and opaque, and the Descemets folds.A round ulcer was seen in the center of the pupil, with a size of approximately 4 mm×4 mm, and the local corneal tissue in the center of the lesion was defective, thin, with gray and white opacities around it and at its base (**Figure 1**). The anterior chamber was moderately deep, mutton-fat keratic precipitates, pale-yellow empyema was observed (2 mm), the iris details was unclear, and the visibility of the posterior segment was poor.

**Figure 2:**
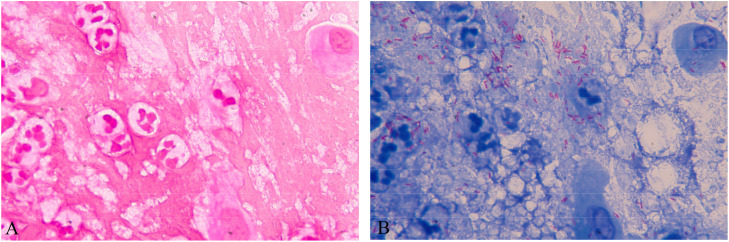
**(A)** Gram-stained corneal scrapings showing unstained “ghost” bacilli. **(B)** On Ziehl–Neelsen counterstaining, the same part showed “ghost” bacilli with red color, which were acid-fast bacilli.

Due to the patient’s long course of disease and repeated disease, the corneal ulcer was grey and turbid focus in appearance, so fungal keratitis was likely. Empirical antifungal (customized voriconazole drops 0.02%) treatment was administered first, while corneal scrapings were sent for examination, all of which were negative. After several days of treatment, the patient’s symptoms were not alleviated but even worsened, suggesting that the clinical judgment was wrong, so the corneal scraping was sent again for examination, and the Gram stain was negative for fungi. Unstained rod-shaped microorganisms were seen in the background (**Figure 2A**). By Ziehl–Neelsen counterstaining, the same site showed acid-fast bacilli (**Figure 2B**). It was suspicious tuberculosis or nontuberculous mycobacterial keratitis. The DNA microarray chip method finally confirmed *Mycobacterium avium* infection. Moxifloxacin eye drops were administered for once an hour, sodium hyaluronate eye drops were given for twice an hour, and rifapentine 0.45 twice daily, ethambutol 0.75 once daily, moxifloxacin 0.4 once daily, and clarithromycin 0.5 once daily were given orally. After 10 days of treatment, the patient’s vision wasn’t change, the eye pain and photophobia were relieved, and the size of the ulcer was the same as before.

**Figure 3:**
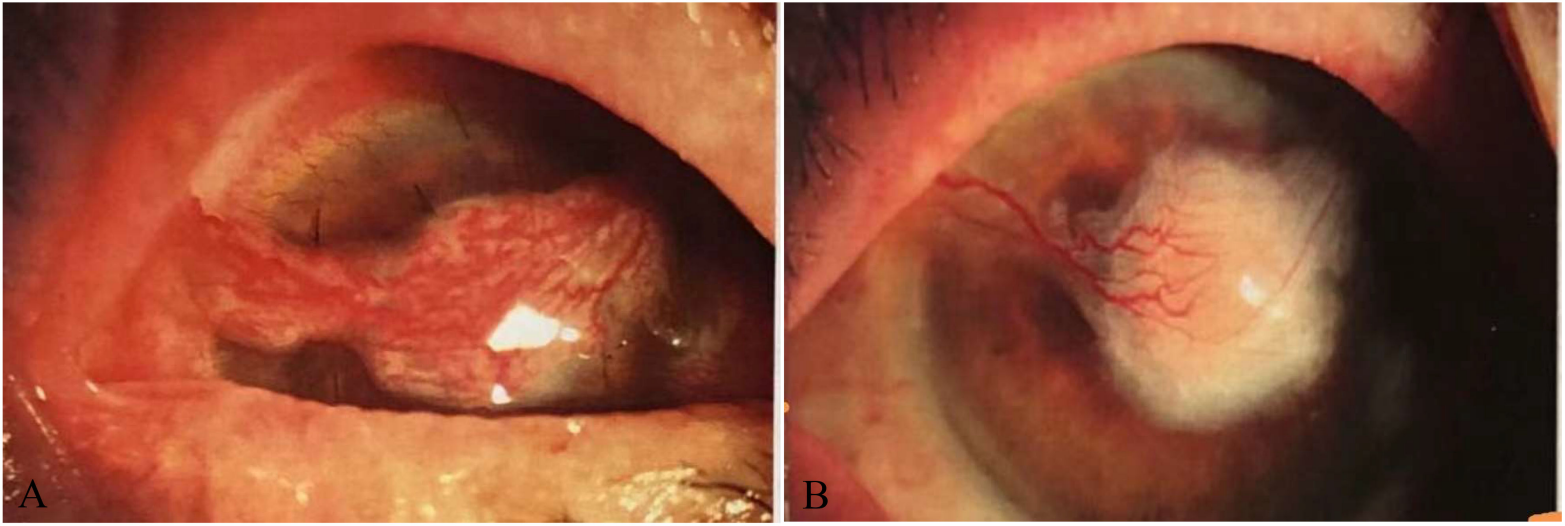
**(A)** Anterior chamber irrigation of the right eye, and autologous conjunctival flap covering the cornea 13 days after surgery. **(B)** Right anterior chamber irrigation, and autologous conjunctival flap covering the cornea 49 days after surgery.

Anterior chamber irrigation with compound electrolyte eye rinse of the right eye plus autologous conjunctival flap covering was performed under general anesthesia, and moxifloxacin eye drops were administered postoperatively sixth daily, atropine sulfate ophthalmic gel once daily, vancomycin eye drops forth daily, and rifapentine 0.45 twice daily, ethambutol 0.75 once daily, moxifloxacin 0.4 once daily, and clarithromycin 0.5 once daily orally. Thirteen days after the operation, the symptoms were relieved, the conjunctiva of the right eye had mixed congestion for the better, and the nasal conjunctiva and its underlying connective tissue had abnormal hyperplasia, invading the corneal limbus by approximately 2 mm. The grafted bridge conjunctival flap was well covered (**Figure 3A**), the anterior chamber was moderately deep, and the condition of the other eye was unclear. The corneal ulcer healed 49 days after the operation (**Figure 3B**), and the combined systemic treatment was continued for half a year.

The main laboratory inspection process and results are shown in **Table 1**.

**Table 1:** Main Laboratory Inspection.

Time	Sample Type	Submission Project	Results
June 3, 2022	Corneal scraping film	Cytological examination of body fluids	Moderate increase in white blood cells, classification: N87% M11% L2%*, epithelial cells (+)
	Corneal scraping film	Gram staining for fungi	No fungi detected, “Ghost” bacteria found
	Corneal scraping film	Add acid fast staining	Detect acid fast staining positive bacteria (+++)
June 3, 2022	Sputum	Mycobacterium tuberculosis nucleic acid	<500 gene copies
June 3, 2022	Sputum	Mycobacterium tuberculosis smear	Not detected
June 4, 2022	Sputum	Mycobacterium tuberculosis smear	Not detected
June 5, 2022	Sputum	Mycobacterium tuberculosis smear	Not detected
June 6, 2022	Blood	T cell detection and interpretation of tuberculosis infection	Positive
June 6, 2022	Eye secretions	Mycobacterium tuberculosis nucleic acid	<500 gene copies
June 6, 2022	Eye secretions	Mycobacterium tuberculosis nucleic acid	<500 gene copies
June 6, 2022	Eye secretions	Bacterial culture	72 hours without bacterial growth
	Eye secretions	Bacterial culture	Extend to 7 days without bacterial growth
June 14, 2022	Eye secretions	Gram staining for bacteria	Not detected
June 14, 2022	Eye secretions	Gram staining for bacteria	Not detected
June 14, 2022	Eye secretions	Fungal culture	7 days without growth
June 14, 2022	Eye secretions	Acid resistant bacteria	Detected acid fast bacilli positive bacteria (+)
June 14, 2022	Eye secretions	Intracellular Mycobacterium Culture	Prolonged to 1 month without growth
June 15, 2022	Eye secretions	DNA microarray chip Method^**^	Mycobacterium avium (+)
June 16, 2022	Blood	T cell detection and interpretation of tuberculosis infection	Positive

^*^N(neutrophile), L(lymphocyte), M(monocyte).

^*^**Mycobacteria Identification Array Kit.

Our patient also underwent other laboratory tests, including whole blood cell analysis: increased eosinophils; Blood biochemistry, coagulation five items, C-reactive protein: no obvious abnormalities; Hepatitis B: hepatitis B virus surface antigen positive, hepatitis B virus e antigen positive, hepatitis B virus core antibody positive; Hepatitis C virus antibody, Treponema pallidum antibody, HIV: negative. Chest Computer Tomography(CT): slight chronic inflammation in both lungs. No congenital or acquired immunodeficiency, no MAC infection found in other areas.

## Discussion

*Mycobacterium avium* keratitis usually has a long time from infection to onset, with reported cases of 21 months (Knapp et al., 1987), 3-4 years (Tyagi et al., 1999), and 4 years (Ko et al., 2017), so it is easy to misdiagnose. Our patient was an elderly woman who was accidentally injured by an agricultural tool in 2005. Four years later, she was diagnosed with keratitis in the right eye complicated by cataract due to her recurrent redness and pain in the right eye with blurred vision. We retrospectively found that the patient’s ocular trauma history, onset time, and clinical symptoms were similar to those reported in the above papers. This led us to speculate that the patient was infected with *Mycobacterium avium* at that time or that the presence of traumatic corneal erosion caused coinfection later. She received levofloxacin, tobramycin, and dexamethasone eye drops, ganciclovir eye drops, and recombinant basic fibroblast growth factor eye drops as treatment in 2009. The symptoms were alleviated but not completely eliminated. The disease recurred, so repeated medication and antifungal drug treatment were administered.

Corticosteroids and broad-spectrum antibiotics have been applied to alleviate infection symptoms in patients with NTM keratitis until the next recurrence, which may mask clinical symptoms and hinder conclusive laboratory testing (John et al., 1992). Mycobacteria are nonflagellated and nonmotile bacteria that mainly infiltrate the paracentral area of the cornea (Martínez et al., 1999). In a cohort of 24 patients with NTM keratitis, histopathology from steroid-treated patients showed mild inflammation despite the abundance of organisms, in contrast to the denser infiltration seen in specimens from patients who did not receive topical steroid (Ford et al., 1998). The corneal ulcer foci of our patient’s right eye were obvious, and the anterior chamber was pale yellow with empyema. In the cytologic examination of the eye scrapings, the bacteria were dense, and the total number of infiltrating leukocytes was slightly to moderately high (**Figure 2**), which may have been caused by the patient’s long-term and multiple medications.

**Figure 4:**
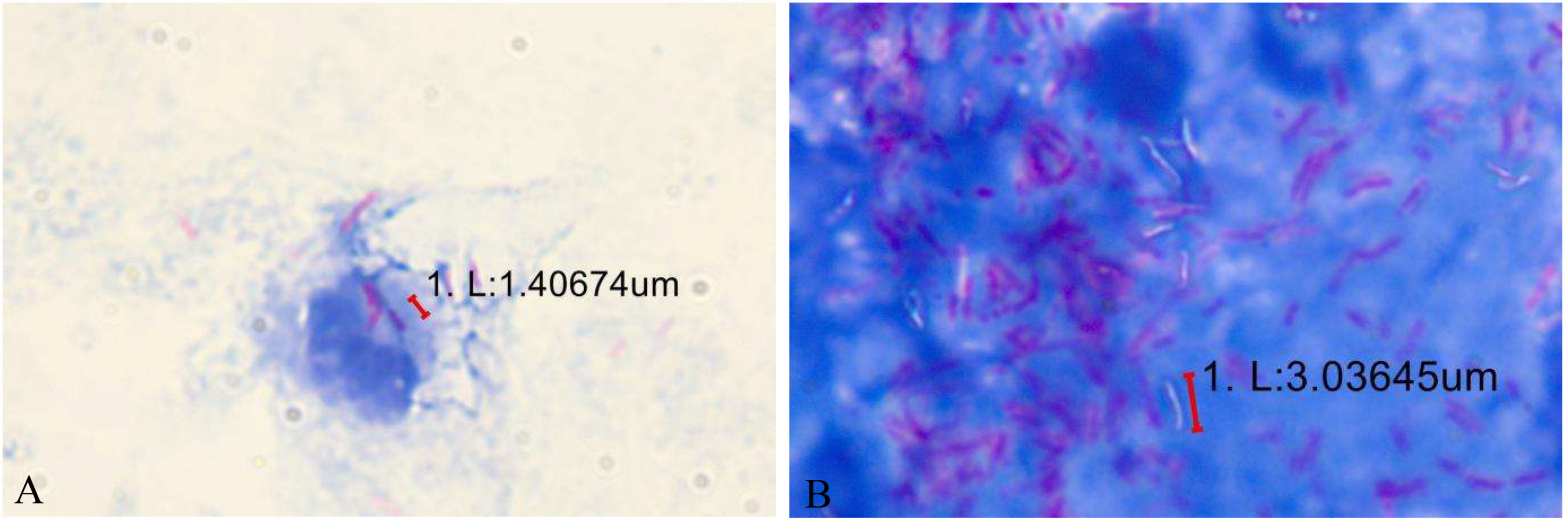
**(A)** Some acidfast bacteria are engulfed inside the cells. **(B)** After Ziehl–Neelsen counterstaining, most of this patient’s samples were positive for acid-fast bacteria, while a small part remained unstained “ghost bacilli”.

The diagnosis of this case mainly relied on Ziehl–Neelsen staining to detect acid-fast bacilli. We finally confirmed *Mycobacterium avium* infection with a DNA microarray chip. The laboratory detection of eye infections caused by Mycobacterium avium still faces several challenges. Compared with fast-growing mycobacteria, the culture of slow-growing mycobacteria is particularly difficult, requiring 2-3 weeks or more to form colonies visible to the naked eye on solid media, and some require special media. For example, *Mycobacterium avium* subsp. *paratuberculosis* requires the addition of mycobactin to the medium. Our patient directly smeared and found acidfast bacteria, and intracellular phagocytosis was observed (**Figure 4A**). However, multiple bacterial cultures were conducted, and they did not grow for 72 hours, 7 days, and 1 month.Fluorescence staining is recommended for microscopic examination, and the positive rate of Ziehl Nielsen staining is relatively low. Unlike *Mycobacterium tuberculosis*, NTM, especially fast-growing ones, are not resistant to acid-alcohol decolorization and are prone to false-negative results. After Ziehl–Neelsen counterstaining, most of this patient’s samples were positive for acid-fast bacteria, while a small part remained unstained “ghost bacilli” (**Figure 4B**). Next Generation Sequencing(NGS) and Polymerase Chain Reaction(PCR) are helpful for early identification of these microorganisms. This method identifies bacteria at the species level by analyzing differences in the composition of homologous DNA sequences. It is currently the gold standard for bacterial species identification but cannot further differentiate NTM to the subspecies level.

The treatment of NTM keratitis is also difficult, as it has traditional antibiotic resistance and a slow treatment response. For patients with no significant alleviation with conservative treatment, surgery is effective and can better eradicate infection and promote ulcer healing. The current patient underwent anterior chamber irrigation of the right eye, and autologous conjunctival flap covering. Her condition was alleviated 13 days after the operation, and the corneal ulcer healed on Day 49 (**Figure 3B**).

In conclusion, we report a case of *Mycobacterium avium* keratitis, the slow growth of *Mycobacterium avium*, delayed symptoms caused by low-grade inflammation, other complex eye diseases such as blurred vision and cataracts caused by advanced age, difficulties in laboratory culture, and recurrence and repeated use of medication were the main reasons for the prolonged failure to clarify the etiology of this case. The laboratory first discovered the unstained “ghost bacilli” in the background during fungal examination, and after verification of Mycobacterium, it was reported to the clinic. Morphological examination provides important clues for the initial discovery of pathogenic bacteria, showing the importance of mycobacterial morphology under Gram staining and Wright’s Giemsa staining. Acid fast staining and Deoxyribonucleic Acid(DNA) microarray chip method can be used as indicators for rapid diagnosis of Mycobacterium species.

## Data availability statement

The raw data supporting the conclusions of this article will be made available by the authors, without undue reservation.

## Ethics statement

Written informed consent was obtained from the individuals for the publication of any potentially identifiable images or data included in this article. Written informed consent was obtained from the participant/patient(s) for the publication of this case report.

## Author contributions

XC: Data curation, Writing – original draft. HW: Writing – review & editing. KW: Methodology. SL: Resources. BL: Investigation. ML: Validation.
